# *CAS*-viewer: web-based tool for splicing-guided integrative analysis of multi-omics cancer data

**DOI:** 10.1186/s12920-018-0348-8

**Published:** 2018-04-20

**Authors:** Seonggyun Han, Dongwook Kim, Youngjun Kim, Kanghoon Choi, Jason E. Miller, Dokyoon Kim, Younghee Lee

**Affiliations:** 1 Department of Biomedical Informatics, University of Utah, University of Utah School of Medicine, Salt Lake City, UT 84108 USA; 2 University of Utah, School of Computing, University of Utah, Salt Lake City, UT 84108 USA; 3 Department of Biomedical & Translational Informatics, Geisinger Health System, Danville, PA 17822 USA; 4 The Huck Institutes of the Life Sciences, Pennsylvania State University, University Park, PA 16082 USA

**Keywords:** Alternative splicing, Cancer, Genomics, Methylation, miRNA, SNP, mRNA regulation

## Abstract

**Background:**

The Cancer Genome Atlas (TCGA) project is a public resource that provides transcriptomic, DNA sequence, methylation, and clinical data for 33 cancer types. Transforming the large size and high complexity of TCGA cancer genome data into integrated knowledge can be useful to promote cancer research. Alternative splicing (AS) is a key regulatory mechanism of genes in human cancer development and in the interaction with epigenetic factors. Therefore, AS-guided integration of existing TCGA data sets will make it easier to gain insight into the genetic architecture of cancer risk and related outcomes. There are already existing tools analyzing and visualizing alternative mRNA splicing patterns for large-scale RNA-seq experiments. However, these existing web-based tools are limited to the analysis of individual TCGA data sets at a time, such as only transcriptomic information.

**Results:**

We implemented *CAS*-viewer (integrative analysis of **C**ancer genome data based on **A**lternative **S**plicing**)**, a web-based tool leveraging multi-cancer omics data from TCGA. It illustrates alternative mRNA splicing patterns along with methylation, miRNAs, and SNPs, and then provides an analysis tool to link differential transcript expression ratio to methylation, miRNA, and splicing regulatory elements for 33 cancer types. Moreover, one can analyze AS patterns with clinical data to identify potential transcripts associated with different survival outcome for each cancer.

**Conclusions:**

*CAS*-viewer is a web-based application for transcript isoform-driven integration of multi-omics data in multiple cancer types and will aid in the visualization and possible discovery of biomarkers for cancer by integrating multi-omics data from TCGA.

**Electronic supplementary material:**

The online version of this article (10.1186/s12920-018-0348-8) contains supplementary material, which is available to authorized users.

## Background

Alternative splicing (AS) is important to our understanding of cancer biology. There are multiple mechanisms by which AS plays a role in cancer, for instance cancer-specific transcript isoforms can be generated [1] or the ratio between mRNA isoforms can be disrupted [2]. Epigenetic factors such as DNA methylation and miRNAs are not only distinct molecular markers of various cancers [3], but methylation and miRNAs are mechanistically linked to the splicing mechanism [4, 5]. Therefore, in order to comprehensively understand the basic principles of mRNA expression patterns in cancer, it will be important to investigate AS with respect to epigenetic factors.

The Cancer Genome Atlas (TCGA) is a comprehensive resource for cancer genomic studies and precision medicine. TCGA has produced a number of multi-omics level data including: transcriptome-wide expression, genetic variants, DNA methylation, miRNA, and clinical information for 33 cancer types. There are existing web resources that allow one to explore, analyze, and visualize TCGA data, including cBioPortal [6], FireBrowse [7], Vials [8] and SpliceSeq [9]. Most of these existing resources are available for exploring alternative mRNA splicing patterns but it is limited to transcriptome based visualization alone. MEXPRESS is a well-designed web-based tool for easy visualization and analysis of multi-layer of omics data - TCGA expression, DNA methylation, and clinical data [10] but lacks the ability to explore alternative mRNA splicing patterns.

In this study, we implemented *CAS*-viewer, offering a set of AS-guided analysis tools for transcripts, miRNAs, DNA methylation, and clinical data from TCGA and SNPs. *CAS*-viewer has several important features. *CAS*-viewer allows users to analyze how different isoforms are associated with DNA methylation in exonic and intronic regions and miRNAs in the 3’ UTR. *CAS*-viewer also correlates the expression ratio with clinical data of interest. It is easy to navigate AS isoforms by using an intron scaling bar, allowing easy conversion between genomic and transcript views. CAS-viewer also provides functional annotations of SNPs by summarizing their co-occurrence with splicing regulatory elements (SREs). Taken together, a tool that is able to integrate AS, expression and epigenetic features, along with clinical data from TCGA provides new ways of conceptualizing how the molecular mechanisms behind cancer can be studied.

## Methods

### Data

***TCGA data***: We compiled level 3 data on transcripts, miRNA expression, DNA methylation, and clinical data for 33 cancer types from the TCGA Genomic Data Commons (GDC) data portal (https://portal.gdc.cancer.gov/). The details for each cancer type and the number of cases in the 33 cancers are summarized in Additional file 1: Table S1. For AS gene model, we used the genomic positions of exons and introns of all transcripts for each gene of a total of 20,465 genes obtained from the Generic Annotation Format (GAF) file, based on the hg19 reference downloaded from the GDC. The GAF file is the same used in the TCGA RNA-seq analysis. We also used the same genome builder for genomic positions of DNA methylation, miRNA targets, and SNPs.

***miRNA target sites***: The miRNA target sites in the 3’ UTR were compiled by integration of three miRNA target databases: 1) miRTarBase [11], which is based on experimentally validated miRNA targets; 2) TargetScan (Release 7.0) [12], which is based on conserved complementarity between targets of miRNAs and mRNAs; and 3) MicroRNA.org, which is based on the miRanda algorithm [13]. First, we obtained all pairs between mRNAs and miRNAs with the miRTarBase database. Second, we obtained the targeted genomic coordinates in paired mRNAs using TargetScan and MicroRNA.org. Then, we identified the 3’ UTR regions that each mRNA binds to according to the Ensembl reference information (release version 75, downloaded from http://ftp.ensembl.org/pub/release-75/gtf/homo_sapiens/, September 2016), comprising 322,389 unique pairs between 2649 mRNAs and 14,894 genes.

***SRE SNP***: To perform a genome-wide scan of SNPs affecting splicing regulatory elements (SREs), we obtained all SNPs and their genomic locations from the VCF files of each chromosome, which were downloaded from the 1000 Genomes Project [14]. The SNPs were divided into two groups, intronic SNPs and exonic SNPs, according to their functional classification. The genome-wide identification of SNPs affecting SREs have been previously described [15]. Using the set of predicted hexameric SRE motifs, including exonic splicing enhancers (ESEs), exonic splicing silencers (ESSs), and intronic splicing enhancers (ISEs) published by Burge and colleagues [16], all potential SRE sites that matched perfectly with any of these hexamers in the entire intragenic region using the twoBitToFa program for hg19 were identified [17]. A total of 4,955,866 SRE SNPs, comprising 527,138 SNPs in ESEs, 196,969 in ESSs, and 4,328,005 in ISEs were annotated.

### Statistical methods

***Calculation of differentially expressed ratio between two groups of transcripts***: For calculating the differential expression ratio between transcripts, we used “Percent spliced in” (PSI) between transcripts that were grouped by users according to the presence or absence of alternative splicing events (i.e., exon skipping, retained introns, or 5′ and 3′ splice sites). PSI is an estimation that uses the FPKM (fragment per kilobase per million fragments) values of transcripts from TCGA RNA-seq data. First, transcripts from a single gene were separated into two groups: one group includes transcripts with a certain splicing events, for example, alternatively spliced exon, and the other contains transcripts without the spliced exon. Each group’s FPKM is summed, and for the two groups, PSI (Ψ_*t*_) is estimated using the *IVAS*, a Bioconductor package for alternative splicing patterns, as follows [18]:

$$ \mathrm{F}\left({\Psi}_t|\right)={\sum}_{i=1}^n{X}_{\mathrm{it}}/\left({\sum}_{s=1}^n{X}_{\mathrm{st}}+{\sum}_{i=1}^n{X}_{\mathrm{it}}\right) $$

Here, *X*_*it*_ and *X*_*st*_ are the expression of transcripts, including and skipping an alternatively spliced exon, respectively. With this PSI value, *CAS*-viewer performs a correlation test with clinical data, methylation level, and miRNA expression as described in the following section.

***Correlation of PSI with clinical data*:** *CAS*-viewer performs a Kaplan-Meier survival analysis using *survival,* which is an R package [19]. First, we divide the cases into two groups by high and low PSI values, using K-means clustering for each group of cases, defined by a user. For the high-PSI and low-PSI cases, we calculate differential survival outcomes using “vital status” and “date to death” information.

***Correlation of PSI with methylation and miRNA in the two groups of cases****:* For each of the two groups of cases defined by the user, we perform linear regression to correlate PSI with methylation levels and miRNA expression. Comparison of differential methylation and miRNA between the two groups of cases is performed using Welch two-sample t-test [20] (the *t.test* function in R package).

***Website***: The web interface of *CAS*-viewer was developed with JavaServer Pages (JSP) and JavaScript.

## Results

*CAS-*viewer is currently available at http://genomics.chpc.utah.edu/*cas*/. On the landing page of *CAS*-viewer, a user can search for an AS gene with a keyword using the HUGO approved gene symbol, DNA methylation cgid, miRNA id, and SNP rs number. The next page returns the searched gene(s) whose intragenic region (i.e., defined as “the transcribed gene region” from the start to end of the transcript) matches with the genomic location of an entered keyword. Clicking a gene links the user to the main page composed of three components: 1) AS Transcript Navigator: for browsing AS transcript isoforms along with methylation sites, miRNA binding sites, and SNPs; 2) Option: select transcripts and cancer cases of interest, based on AS events (i.e., exon skipping, intron retention, and 5′ and 3′ splice site) and clinical information, respectively; and 3) Output: shows the results for differential expression between selected transcripts (i.e., PSI; see Methods), its association with selected clinical features, DNA methylation level, targeting miRNA expression, and SNPS located in SREs.

**Fig. 1:**
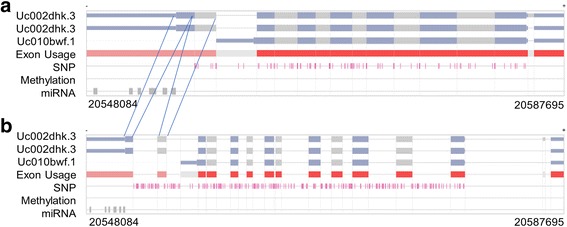
AS Transcript Navigator. **a** Screen shot of transcript models for the ACSM2B gene as an example. This gene is transcribed into three mRNAs, comprising 16 distinct exons in total. Blocks and lines indicate exons and introns. Among exon blocks, the thicker blocks are coding regions (CDS), and thinner blocks are untranslated regions (UTRs). AS Transcript Navigator contains tracks of exon usage, SNPs, Met, and miRNAs within the defined transcribed region of a gene. Exon usage is composed of the representative exons, clustered according to the genomic location of overlapping exons. The SNP, Met, and miRNA tracks represent their respective locations. Transcripts in the mRNA coordinate when the intron scale is 0% (**a**), and both exons and introns are equivalent in the genome browser when the intron scale is 100% (**b**)

***AS Transcript Navigator***: All transcript isoforms of the selected gene are illustrated with exons and introns, which are represented with rectangles and lines, respectively. Among exon blocks, thicker blocks are the coding region (CDS), and thinner blocks are untranslated regions (UTRs). SRE SNPs, methylations, and miRNAs that exist within the defined transcribed region of a gene are visualized accordingly (Fig. 1a). The two useful functions in AS Transcript Navigator are exon usage track and intron scaling.

“Exon Usage”, the last transcript track, is composed of the representative exons that are defined by the clustering of overlapping exons (Fig. 1a). Exons are clustered according to genomic location to find overlapping exons. Then, we create “representative exons,” which is essentially a concatenation of the longest exons in each exon cluster. Exons whose length differs from the representative exon can be easily recognized to be alternatively spliced (i.e., 5′ and 3′ splice sites and intron retention). The color on the exon represents the skipping frequency of each exon; the lighter the color, the more frequently it is skipped. The user can also see the same information through the mouse over pop-up for each exon that shows how many transcripts miss the given exon.

An intron scaling bar was implemented to allow seamless transition from the genome browser (unspliced/pre-mRNA) to the transcript viewer (spliced/mature transcript), combining advantages of the two viewers. An intron scale of 0% shows transcripts in the mRNA coordinate that more easily indicate splicing features (Fig. 1a). An intron scale of 100% makes the viewer equivalent to the genome browser, which is most convenient to specify the genomic features in introns (Fig. 1b). Since introns are much (> 5) longer than exons in eukaryotic genomes, most space in the genome browser is assigned for introns, and AS events, such as alternative splice sites, are not easily recognized. Therefore, scaling introns to a small value is helpful to show minute variations in exon length, maintaining the exon-intron boundary.

**Fig. 2:**
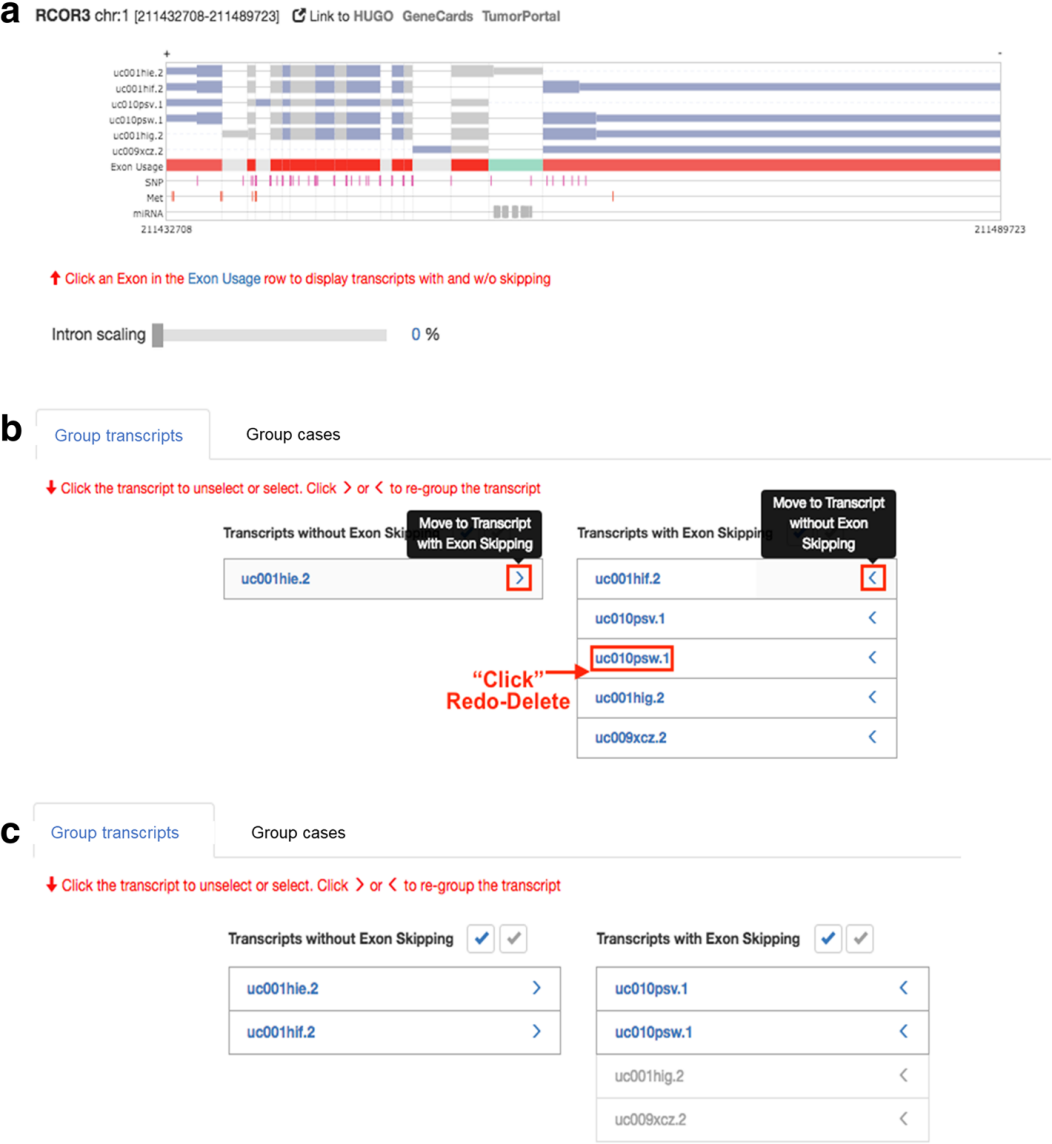
Option: Group transcripts. **a** When the user clicks an exon of interest, the selected exon is highlighted in green. Using re-grouping options, pre-divided transcript groups according to the selected exons (**b**) can be re-grouped with redo-selection within a group, and a transcript can be moved between groups (**c**)

In order to start, the user first selects an exon of interest in AS Transcript Navigator, next going to the “Option” section. The selected exon is highlighted in green (Fig. 2a). AS transcripts and all plots can be downloadable as a PNG and SVG format.

***Option*:** There are two options that enable users to divide transcripts and cancer cases into two groups: Group transcripts and Group cases.

Once the user selects an exon of interest in AS Transcript Navigator, by default, the transcripts are automatically pre-divided into two groups: transcript groups with and without skipping of the selected exon (Fig. 2a). Then, the user can further re-group the transcripts by 1) clicking the transcript id to unselect the pre-selected transcript and 2) clicking “>” or “<” to move the transcript into another group. Fig. 2b and Fig. 2c show the re-grouping options and regrouped transcripts, respectively. As the expression ratio of certain mRNA transcript isoforms are often imbalanced or altered in cancers cell, the ratio of differential expression (denoted “percent spliced in” (PSI)) between the two transcript groups will be calculated and plotted in “Transcript ratio” of the output panel. The PSI value will be used for correlation test with clinical data, methylation data, and miRNA data.

**Fig. 3:**
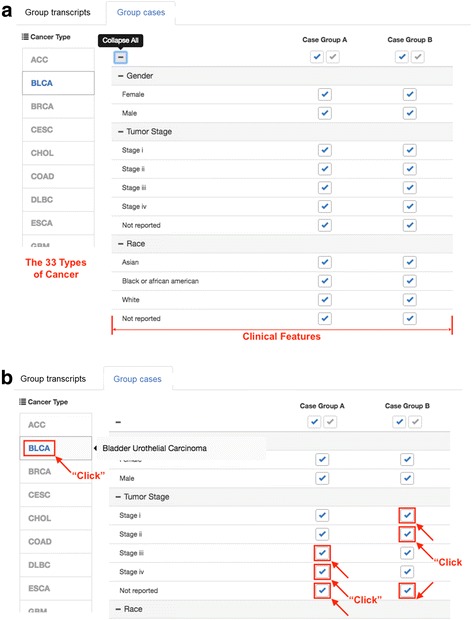
Option: Group cases. **a** In the left panel, the user clicks one cancer type and then clicks clinical features in ‘case inclusion’ option. **b** Within each clinical category, the “OR” option is selected, and between clinical categories, the “AND” option is selected to group a case

The user can select a case according to its clinical information for a cancer type (Fig. 3a). Thirty-three cancer types are available in the option section (Additional file 1: Table S1). The user defines two groups of cases within a cancer type to compare the clinical features to test whether the PSI correlates with expected survival times (Fig. 3b). This feature helps users identify the transcript isoforms (or exons) that are related to the clinical outcome of interest.

***Output***: Output comprises five sections in plotting the results: Transcript ratio, Clinical correlation, Methylation, miRNA, and SRE SNPs.

**Fig. 4:**
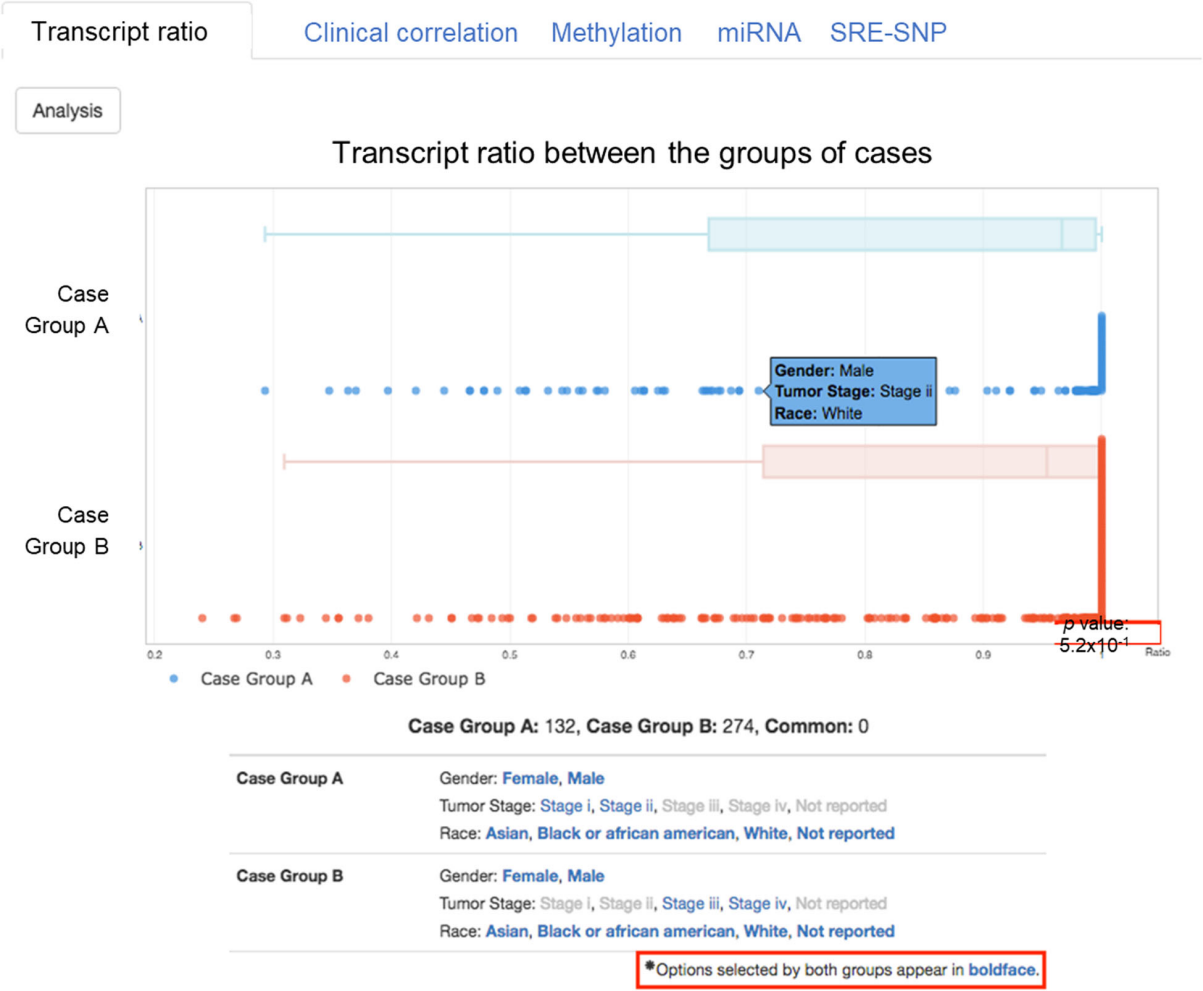
Output of transcript ratio grouped by exon 16 in RCOR3 gene. The x-axis is PSI value, ranging from 0 to 1, and the y-axis is the two groups of cases. The plot shows the mouse over pop-up for each dot, giving detailed clinical information on each case; the *p*-value between differential PSI values between two groups of cases is shown at the bottom right. Under the plot, the table summarizes the number of cases for each group and the common cases in both groups, followed by the selected clinical features for each group

“Transcript ratio” illustrates the results in the boxplot, showing the distribution of PSI values between two groups of cases, which is user-defined in the “Group Cases” Option. The PSI value refers to the differential expression between two groups of transcripts. In Fig. 4, the X-axis is PSI value, ranging from 0 to 1, and the Y-axis is the two groups of cases. The plot shows the mouse over pop-up for each dot, giving detailed clinical information on each case; the *p*-value between differential PSI values between two groups of cases is shown at the bottom right. Under the plot, the table summarizes the number of cases for each group and the common cases in both groups, followed by the selected clinical features for each group.

**Fig. 5:**
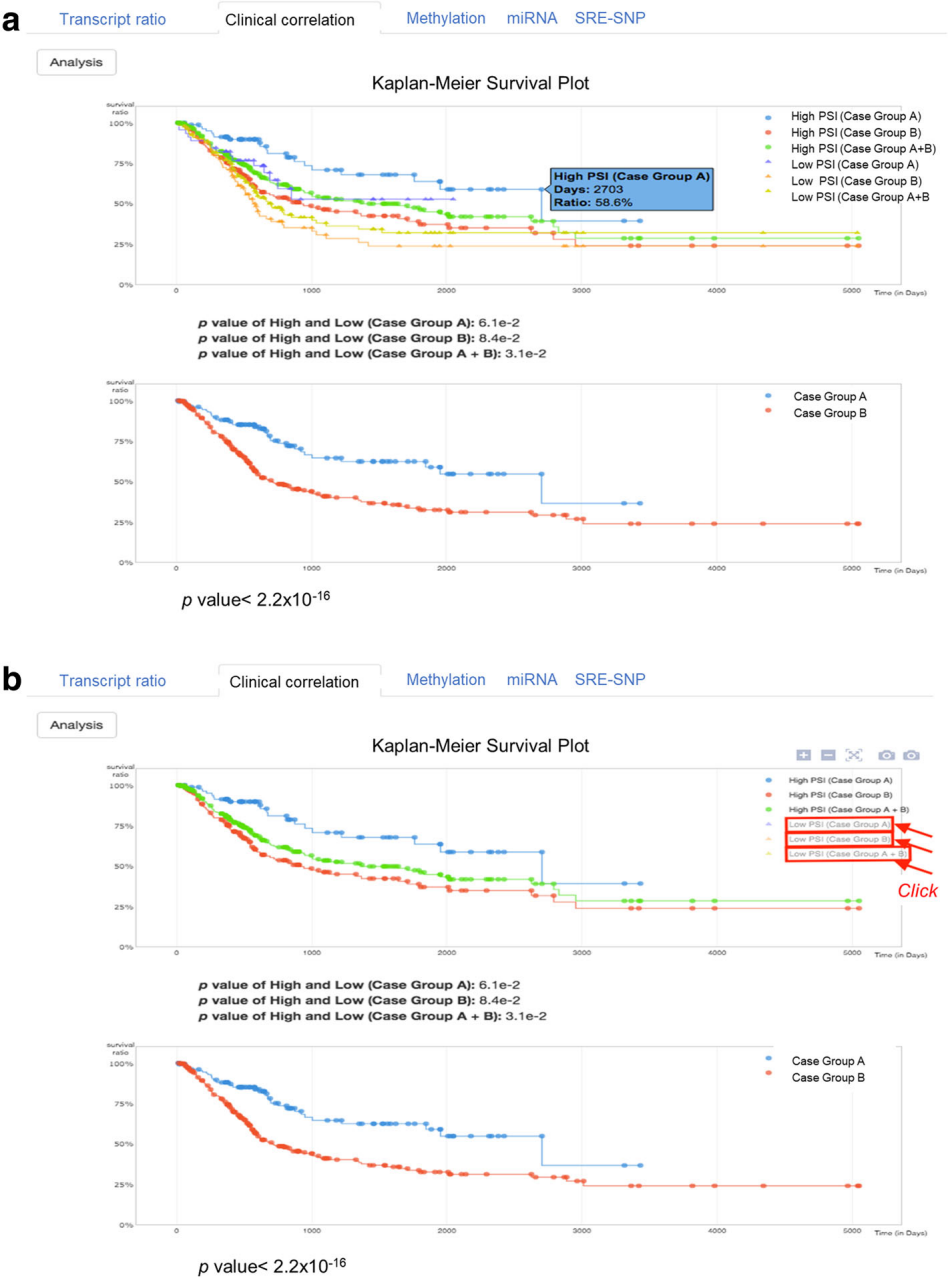
Output of Clinical correlation of exon 16 in the RCOR3 gene. **a** The pair of high- and low-PSI subgroups for each Case Group is tested for significance of the differential survival outcome (**upper panel**) and the survival differences between Case Groups A and B are shown (**bottom panel**). **b** Each legend is clickable, with the corresponding plot appearing and disappearing. The dot for each survival time point displays further information on the mouse over pop-up

“Clinical correlation” summarizes the Kaplan-Meier plots, showing the correlation of PSI values with survival outcomes for three subgroups of cases: Case Group A, Case Group B, and Case Group B with high and low PSI. Then, the pair of high- and low-PSI subgroups for each Case Group is tested for significance of the differential survival outcome (upper panel of Fig. 5a). The X-axis is expected survival time in days, and the y-axis is the survival ratio. In addition, the survival differences between Case Groups A and B are shown (bottom panel of Fig. 5a). In the upper right of the plot, each legend is clickable, with the corresponding plot appearing and disappearing (Fig. 5b). The dot for each survival time point displays further information on the mouse over pop-up.

**Fig. 6:**
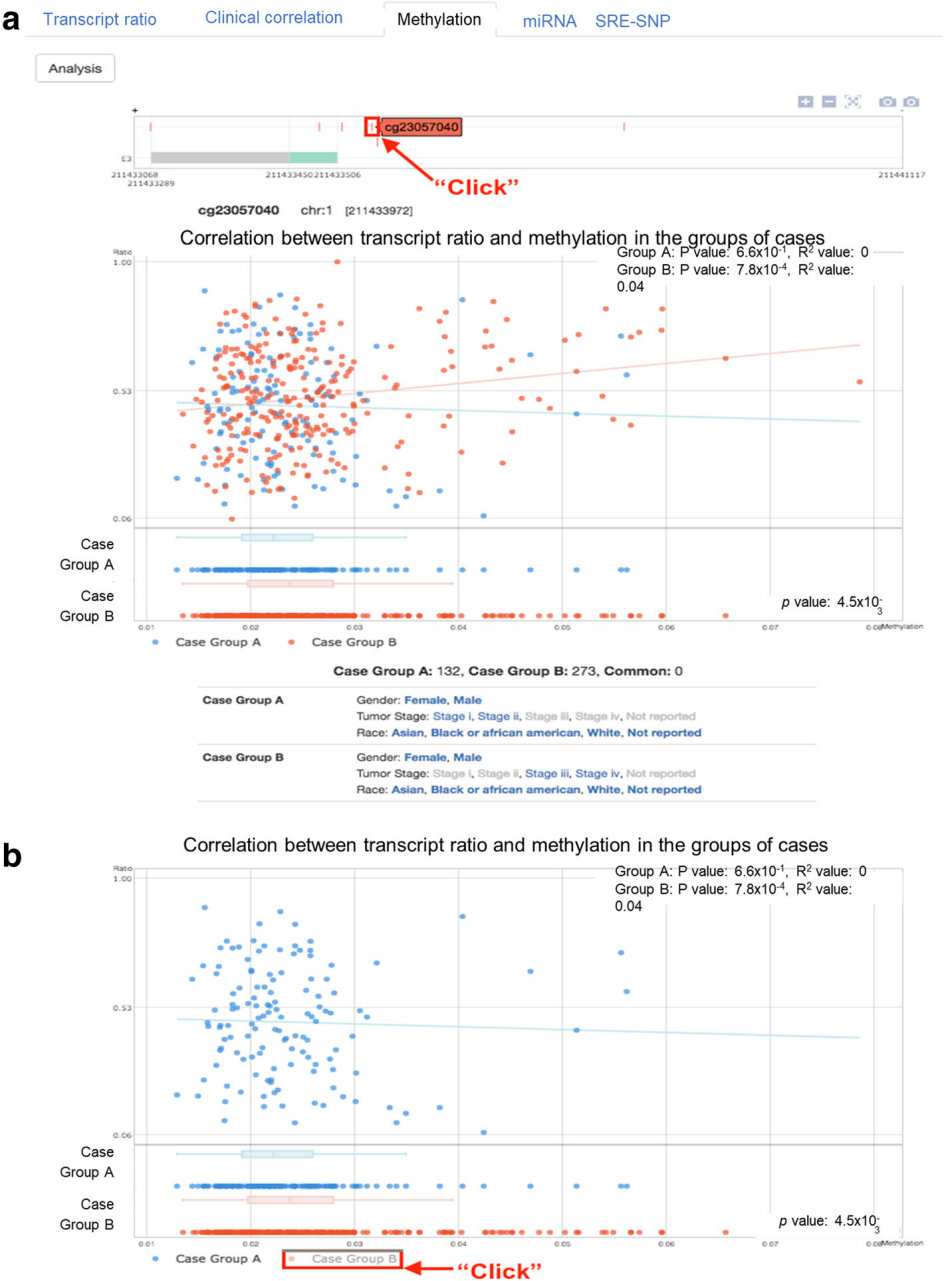
Output of Methylation results for cg23057040 of exon 3 of the RCOR3 gene. **a** The plot summarizes the regression analysis by showing the distribution of cases according to PSI values on the y-axis and methylation status on the x-axis. **b** At the bottom of the plot, the names of groups (i.e., Case Group A and Case Group B) are clickable, appearing and disappearing with the corresponding distribution of cases in the plot area

In “Methylation”, once a user clicks a certain exon in AS Transcript Navigator, this panel automatically displays the zoomed-in view of the selected exon, including the right (downstream) and left (upstream) adjacent introns and all methylation that exists within the displayed genomic region. When a user clicks a point of methylation and the “Analysis” button, the plot summarizes the regression analysis by showing the distribution of cases according to PSI values on the y-axis and methylation status on the x-axis (Fig. 6a). The mouse over pop-up on the dot displays the selected clinical features for a case. If a case is common in two groups of cases, the dot is green in the plot. At the bottom of the plot, the names of groups (i.e., Case Group A and Case Group B) are clickable, appearing and disappearing with the corresponding distribution of cases in the plot area (Fig. 6b).

**Fig. 7:**
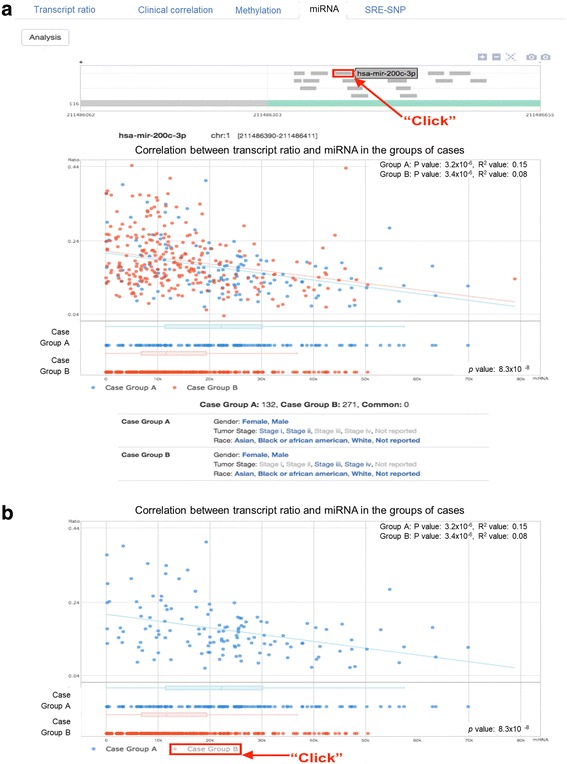
Output of miRNA results for hsa-mir-200c-3p in exon 16 of the RCOR3 gene. **a** The plot summarizes the regression analysis by showing the distribution of cases according to PSI values on the y-axis and miRNA expression on the x-axis. **b** At the bottom of the plot, the names of groups (i.e., Case Group A and Case Group B) are clickable, appearing and disappearing with the corresponding distribution of cases in the plot area

In “miRNA”, once a user clicks a certain exon in AS Transcript Navigator, this panel automatically displays the zoomed-in view of the selected exon and all miRNA binding sites that exist within the displayed genomic region. When a user clicks a miRNA and the “Analysis” button, the plot summarizes the regression analysis by showing the distribution of cases according to PSI values on the y-axis and miRNA expression on the x-axis (Fig. 7a). The mouse over pop-up on the dot displays the selected clinical features for a case. If a case is common in two groups of cases, the dot is green in the plot. At the bottom of the plot, the names of groups (i.e., Case Group A and Case Group B) are clickable, appearing and disappearing with the corresponding distribution of cases in the plot area (Fig. 7b).

**Fig. 8:**
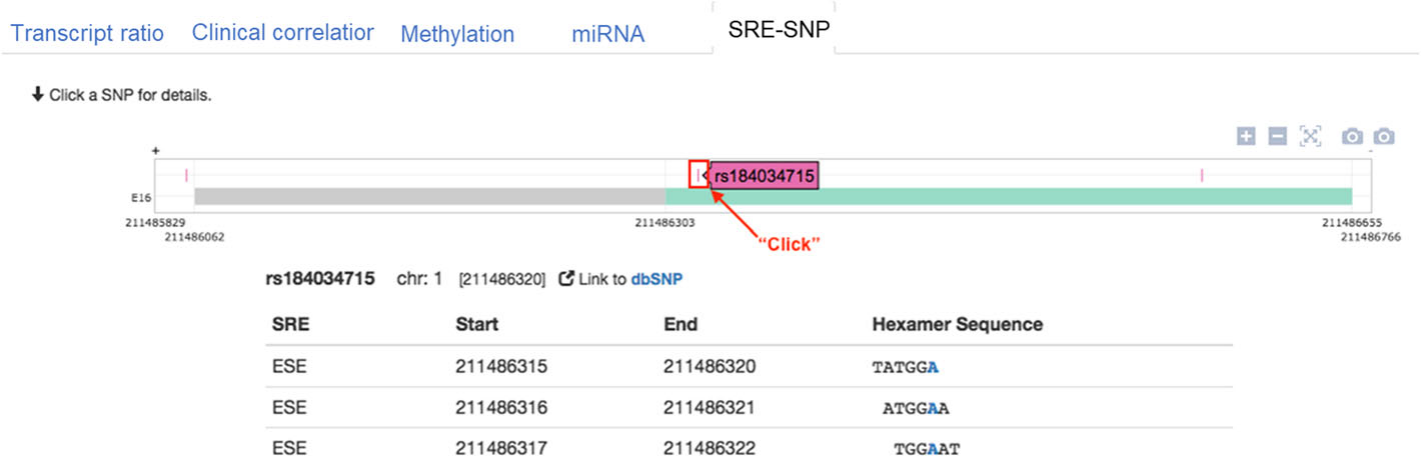
SRE SNP results for rs184034715 in exon 16 of the RCOR3 gene. Hexameric sequences of SREs (i.e., ESE, ESS, and ISE) including SNPs are listed and the blue letters represent SNPs in the hexameric sequences

In “SRE SNP”, the genomic regions showing SRE SNPs for a selected exon comprises the right and left introns adjacent to the exon and selected exon. When one selects an SNP, this component summarizes the hexameric SRE motifs (i.e., ESE, ESS, and ISE) in the SNPs. As an example, for rs184034715, the ‘SRE-SNP’ tab shows the zoomed-in view of the selected exons and introns as defined above with SRE SNPs. Clicking the SNP displays all predicted SRE information, including genomic location and hexameric sequences (Fig. 8).

### Quick start

**Fig. 9:**
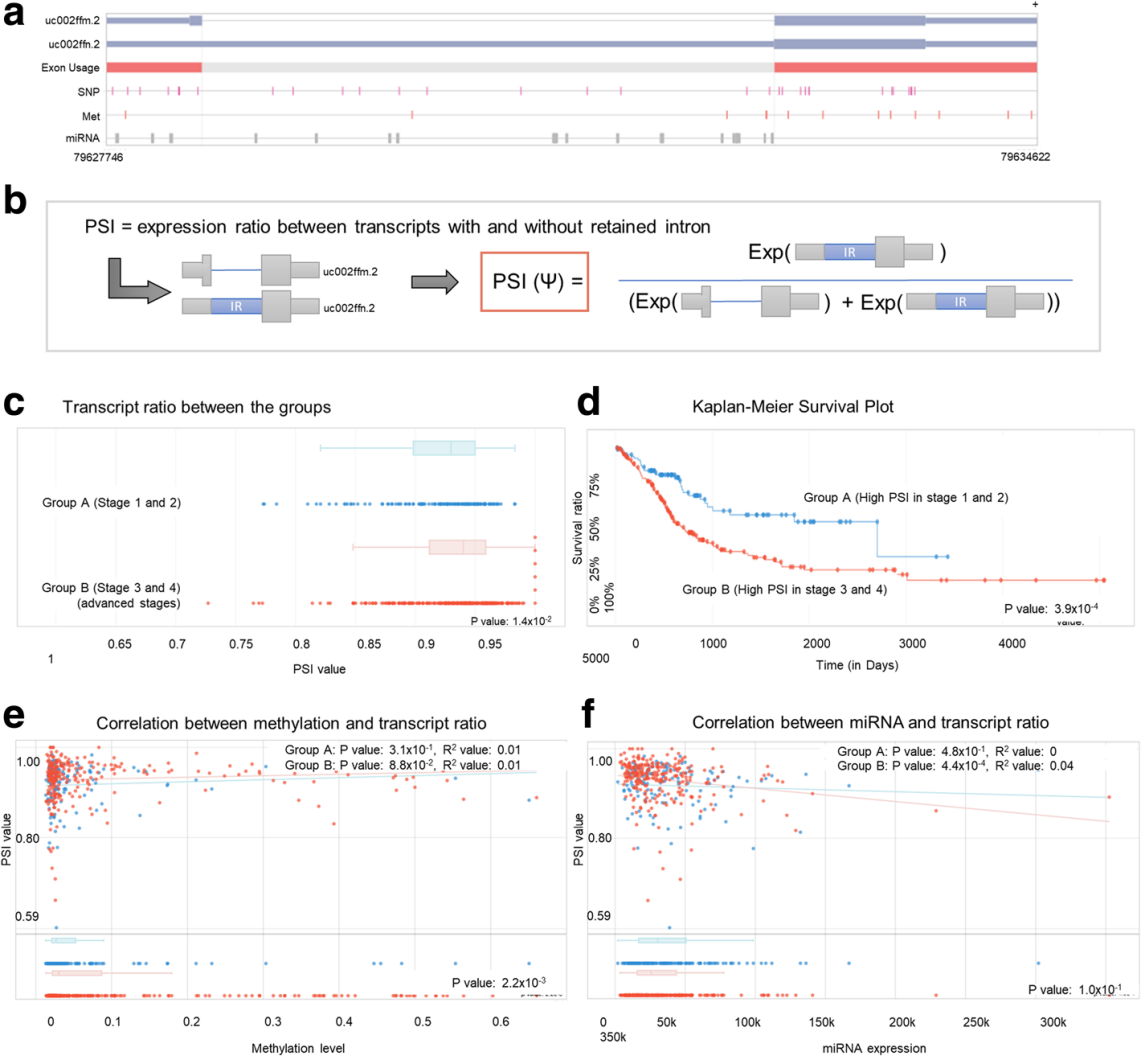
Case study of *MAF*. **a** The *MAF* gene can be alternatively spliced into two mRNAs, one with a retained intron and the other one without a retained intron. **b** PSI refers to the expression ratio between transcripts with and without the retained intron. The higher PSI value, the higher expression of the transcript with retained intron. **c** X-axis represent PSI values. Cases in the Group A are in the blue dots and box plot and cases in the Group B are in the red dots and box plots. **d** Subgroups with high PSI values in the Group A showed better clinical outcome than subgroups with high PSI values in the Group B. **e** X-axis represents methylation level and Y-axis represents PSI values. **f** X-axis represents miRNA expression level and y-axis represents PSI values

In a single gene query, users can visualize the alternative splicing mRNA isoforms with methylation, miRNA, and SRE data and the clinical correlation for cancers. Step 1. Select a gene. Step 2. Select an exon of interest in the AS Transcript Navigator (Fig. 2a). Step 3. Select the transcripts in order to divide them into two groups for calculating the ratio of differential expression (PSI) between the groups of transcripts (Fig. 2b). Step 4. Select a cancer type and patient/case set (Fig. 3a). Step 5. Results tab of the “transcript ratio” shows the scatter and box plots of PSI values for each case by transcript group. The selected transcripts are differentially expressed in two case groups (i.e., two-stage groups in bladder cancer (Fig. 9b-c). Step 6. Results tab of “Clinical correlation” offers the Kaplan-Meier survival plot for all selected case by group (Fig. 9d). Step 7. Results tab of “Methylation” displays the scatter plot of PSI compared with DNA methylation data of a gene across all selected cases by group (Fig. 9e). Step 8. Results tab of “miRNA” displays the scatter plot of PSI compared with miRNA data of a gene across all selected cases by the group (Fig. 9f). Step 9. Results tab of “SRE-SNP” offers SNPs annotated as an SRE (Fig. 8). The details for a user’s manual are described in Additional file 2: CAS-Viewer Quick Start.

### Case study: *MAF*

The key feature of *CAS*-viewer is that it can explore multi-omics data such as methylation, miRNA, and clinical information in the context of transcript isoforms. We presented a case study with *MAF* (MAF BZIP Transcription Factor) gene for bladder cancer as an example, to demonstrate how one can use *CAS*-viewer to gain insight into comprehensive understanding of molecular complexity existing among splicing, methylation, and miRNA, and its clinical correlation. The *MAF* gene is a transcription factor and well-known oncogene as a member of the AP1 superfamily. The *MAF* gene is known to produce two isoforms, uc002ffn.2 (NM_001031804) and uc002ffm.2 (NM_005360) (Fig. 9a and b). uc002ffn.2 isoform includes a retained intron between exon 1 and 2 in the 3’ UTR region, but uc002ffn.2 does not. We first investigated whether expression ratio between the transcripts with and without retained intron (“PSI” value) differs in the stages of bladder cancer. The higher PSI value refers to the higher expression of isoforms with retained intron compared to the isoform without trained intron (Fig. 9a). We selected two groups of cases by bladder cancer stages, Group A consists of cases in stage 1 (*N* = 2) and 2 (*N* = 130) and Group B consists cases in stage 3 (*N* = 140) and 4 (*N* = 134). As shown in Fig. 9c, PSI values in Group B (red dots) were significantly higher than in Group A (blue dots) (*p* = 0.014), suggesting that expression level of transcript isoforms with retained intron were higher in Group B than Group A. Subgroups from the highest quantiles of PSI values in Group B (*N* = 212) showed a poorer clinical outcome (*p* = 0.000393, Fig. 9d) compared to subgroups from the highest qualities of PSI values in the Group A (*N* = 108). It suggests that changed or imbalanced expression ratio between spliced mRNA isoforms of *MAF* may be associated with a worse survival outcome in bladder cancer and be more important for the cases in the advanced stages (3 and 4) rather than cases in the stage 1 and 2 in bladder cancer.

Epigenetic factors affecting gene expression, such as methylation and miRNA, are mechanistically linked to splicing as well. We tested whether the methylation site (cg07870982) and miRNA (hsa-mir-10b-3p) located in the retained intron of MAF are associated with transcript ratio. DNA methylation was not significantly correlated with the transcript ratio in both groups (**upper plot of** Fig. 9e), but DNA methylation level was higher in Group B compared to Group A (*p* = 0.002, **lower plot of** Fig. 9e). Notably, as expected, expression of hsa-mir-10b-3p was reversely correlated with the transcript ratio in Group B only (advanced stage of bladder cancer) (*p* = 0.0004, R^2^ = 0.04, **upper plot of** Fig. 9f) but not Group A, even though hsa-mir-10b-3p were similarly expressed in the two groups (**lower plot of** Fig. 9f). These results suggest that early and late stages of bladder cancer can be differentiated by the ratio between miRNA and target transcript isoform using *MAF* alone.

## Discussion

Several recent studies have introduced web-based tools to visualize and analyze multidimensional cancer genomic data, such as genetic alternations, gene expression, and methylation. As summarized in Table 1, none of these web-based tools provides splicing-based multi-omics data analysis. For example, cBioPortal is a platform used to investigate genetic alterations (i.e. mutations and copy number variants) with gene expression, clinical outcomes or protein structures in cancer, but lacks splicing-driven integration [6]. FireBrowse also summarizes a statistical result across multiple types of omics data (i.e., methylation, miRNA, SNP, and clinical data) in text format but also lacks the ability to explore how splicing is connected to all of these different data types [21]. MEXPRESS is a well-designed web-based tool for visualization and analysis of multi-omics data including gene expression, DNA methylation, and clinical data but the alternative splicing (i.e. transcript isoforms or splicing events) not considered [10]. Such tools are useful to explore multi-omics data at the gene level expression only. Even though there are web-based tools, Vials [8], SpliceSeq [9], TCGASpliceSeq [22], and ISOexpresso [23], that are useful to investigate alternative splicing events or transcript isoforms, these tools are limited to analyze a single-layer of omics data.

**Table 1:** Comparison of the web-based tools for visualizing cancer omics data

	Alternative splicing	Methylation	miRNA	SNP	Clinical data
cBioPortal [6]	x	x	x	o	o
FireBrowse [21]	x	o	o	o	o
MEXPRESS [10]	x	o	x	x	o
Vials [8]	o	x	x	x	x
SpliceSeq [9] and TCGASpliceSeq [22]	o	x	x	x	x
ISOexpresso [23]	o	x	x	x	o
CAS-viewer	o	o	o	o	o

## Conclusions

Here, we have presented *CAS*-viewer, a web-based tool, a splicing-guided integrative analysis tool of multi-layer omics data sets such as RNA-seq, methylation, miRNA, and clinical information on a large number of cases across diverse cancer types. We expect that *CAS*-viewer will be a useful resource for bioinformaticians and non-bioinformaticians who study cancer. Furthermore, *CAS*-viewer will aid in the visualization and possible discovery of biomarkers for cancer by integrating multi-omics data from TCGA.

## Additional files

Additional file 1: **Table S1.** Summary of the 33 cancer types in TCGA and the cases compiled in CAS-viewer. (PDF 24 kb)

Additional file 2: CAS-Viewer Quick Start. (PDF 2182 kb)
