# Supplementary material for: CAS-viewer: web-based tool for splicing-guided integrative analysis of multi-omics cancer data
Source: BMC Med Genomics. 2018 Apr 20;11(Suppl 2):25. doi: 10.1186/s12920-018-0348-8 (PMC5918456; doi:10.1186/s12920-018-0348-8)
Supplement: Supplementary file 1 — Table S1. Summary of the 33 cancer types in TCGA and the cases compiled in CAS-viewer. (PDF 24 kb) [file 12920_2018_348_MOESM1_ESM.pdf]

Supplementary Table 1. Summary of the 33 cancer types in TCGA and the cases compiled in *CAS*-viewer.

Type	mRNA expression	DNA methylation	miRNA expression
**Platform**	**Illumina HiSeq**	HumanMethylation 450K	**Illumina HiSeq**
TCGA-BRCA	1093	783	749
TCGA-HNSC	520	528	483
TCGA-LGG	516	516	510
TCGA-THCA	505	507	502
TCGA-PRAD	497	498	491
TCGA-LUAD	515	458	448
TCGA-UCEC	370 [1]	431	398
TCGA-BLCA	408	412	409
TCGA-STAD	415	396	387
TCGA-LIHC	371	377	369
TCGA-LUSC	501	370	336
TCGA-KIRC	531	319	239
TCGA-CESC	305	307	306
TCGA-COAD	285	297	251
TCGA-KIRP	290	275	286
TCGA-SARC	259	261	256
TCGA-ESCA	184	185	182
TCGA-PAAD	178	184	177
TCGA-PCPG	179	179	178
TCGA-TGCT	150	150	149
TCGA-GBM	154	287	566 [3]
TCGA-THYM	120	124	124
TCGA-SKCM	103	104	97
TCGA-READ	94	98	89
TCGA-MESO	87	87	87
TCGA-ACC	79	80	79
TCGA-UVM	80	80	80
TCGA-KICH	66	66	65
TCGA-UCS	57	57	56
TCGA-DLBC	48	48	47
TCGA-CHOL	36	36	36
TCGA-OV	304	592 [2]	477
TCGA-LAML	123	140	136 [4]
Total	9423	9232	9045

[1] mRNA expression for this cancer type were complied with the data generated by the Illumina GA platform. [2] DNA methylation for this cancer type were complied with the data generated by the HumanMethylation27K BeadChip platform. [3] miRNA expression for this cancer type were complied with the data generated by the Agilent 8 x 15K Human miRNA-specific microarray platform. [4] miRNA expression for this cancer type were complied with the data generated by the Illumina GA platform.
