# Supplementary material for: CAS-viewer: web-based tool for splicing-guided integrative analysis of multi-omics cancer data
Source: BMC Med Genomics. 2018 Apr 20;11(Suppl 2):25. doi: 10.1186/s12920-018-0348-8 (PMC5918456; doi:10.1186/s12920-018-0348-8)
Supplement: Supplementary file 2 — CAS-Viewer Quick Start. (PDF 2182 kb) [file 12920_2018_348_MOESM2_ESM.pdf]

CAS-viewer Quick Start

1. Introduction

1.1 Overview

1.2 Environment

2. Get started

2.1 Landing page

2.2 Keyword search

3. AS Gene Navigator

3.1 AS Gene Viewer

3.2 Exon Usage Track

3.3 Methylation, miRNA, and SRE SNP Track

3.4 Intron scaling

3.5 Select an exon

4. Option

4.1 Group a Transcript

4.2 Group a Case

5. Output

5.1 Transcript Comparison

5.2 Clinical Correlation

5.3 Methylation

5.4 miRNA

5.5 SRE SNP

1. Introduction
1.1 Overview

CAS-viewer (Visualization of Cancer Alternatively Splicing) is a web-based tool representing cancer genomes. It visualizes alternative splicing (AS) gene-level molecular information and its clinical implication. *CAS*-viewer retrieves the datasets compiled with genome-wide data on transcripts, miRNAs, and DNA methylation for 33 cancer types obtained from TCGA data as of September 2016. *CAS*-viewer is a dynamic interface allowing one to scale a length of an intron to transit easily between transcript and genomic views of AS transcripts isoforms. Moreover, one can analyze AS patterns with clinical data, along with DNA methylation or miRNA. It allows one to identify molecular factors contributing to the given clinical features that are correlated with different survival outcomes. Splicing regulatory elements and hexameric DNA sequences are also available for interpreting the functional role of both intronic and exonic SNPs.

1.2 Environment

CAS-viewer is compatible with most types of web browsers in Windows, Linux, and Mac OS. As long as your OS has installed the latest versions of any web browser, you can freely use *CAS*-viewer. There is a list of web browsers and the versions that we tested and are compatible. Please contact with us at issue.leelab@gmail.com if you have any technical issues and questions on using *CAS*-viewer.

CAS-viewer is compatible with any of the web browsers and their versions for Windows, Linux, and Mac as follows:

- Chrome version 56.02924.87 (64-bit)
- Firefox version 52.0.2 (64-bit)
- Internet Explorer 11.953.14393.0 (64-bit)
- Microsoft Edge 38.14393.0.0 (64-bit)
- Safari 10.1 (64-bit)

2. Get Started

2.1 Landing page

CAS-viewer is available at http://genomics.chpc.utah.edu/cas/ (**Figure 1**).

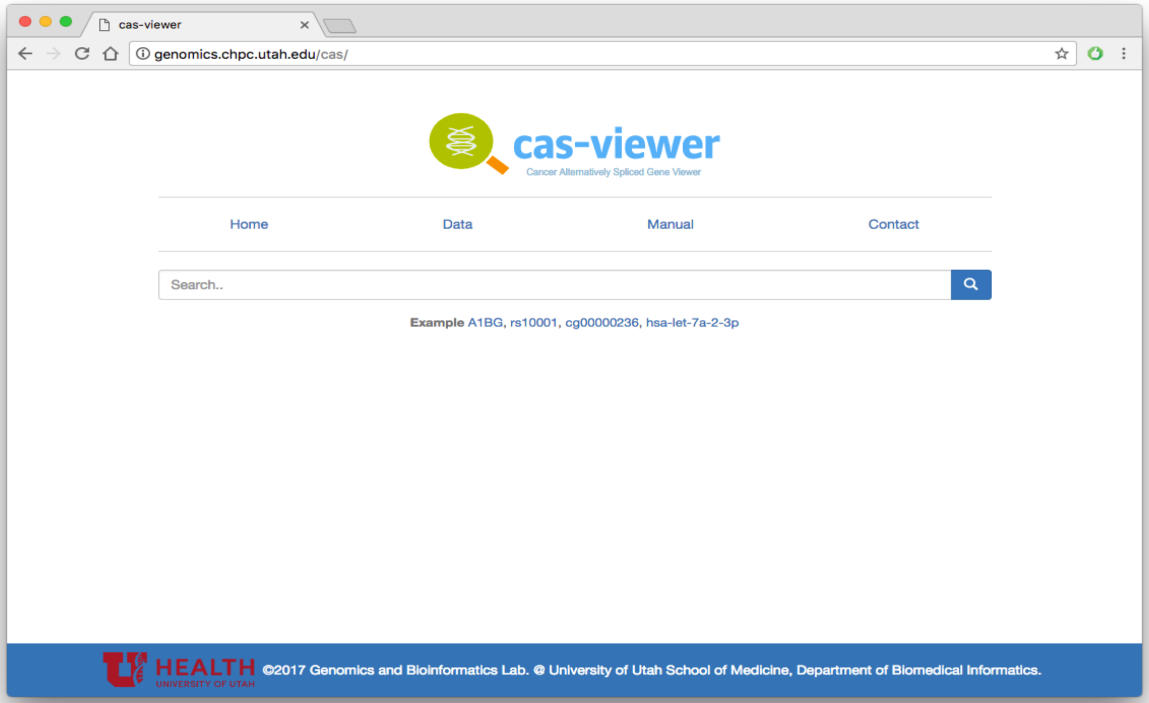

Figure 1. The landing page of CAS-viewer

2.2 Keyword Search

As an input for *CAS*-viewer, the HUGO-approved gene name, SNP (rs number), DNA methylation number (cgID), and miRNA ID are searchable for an alternatively spliced gene: e.g., A1BG, 'rs10001,' 'cg00000236,' and 'hsa-let-7a-2-3p' (**Figure 1**). The next page displays the search results for alternatively spliced genes that match the input keyword (**Figure 2**). For miRNAs, multiple genes can be listed, because one miRNA targets multiple genes.

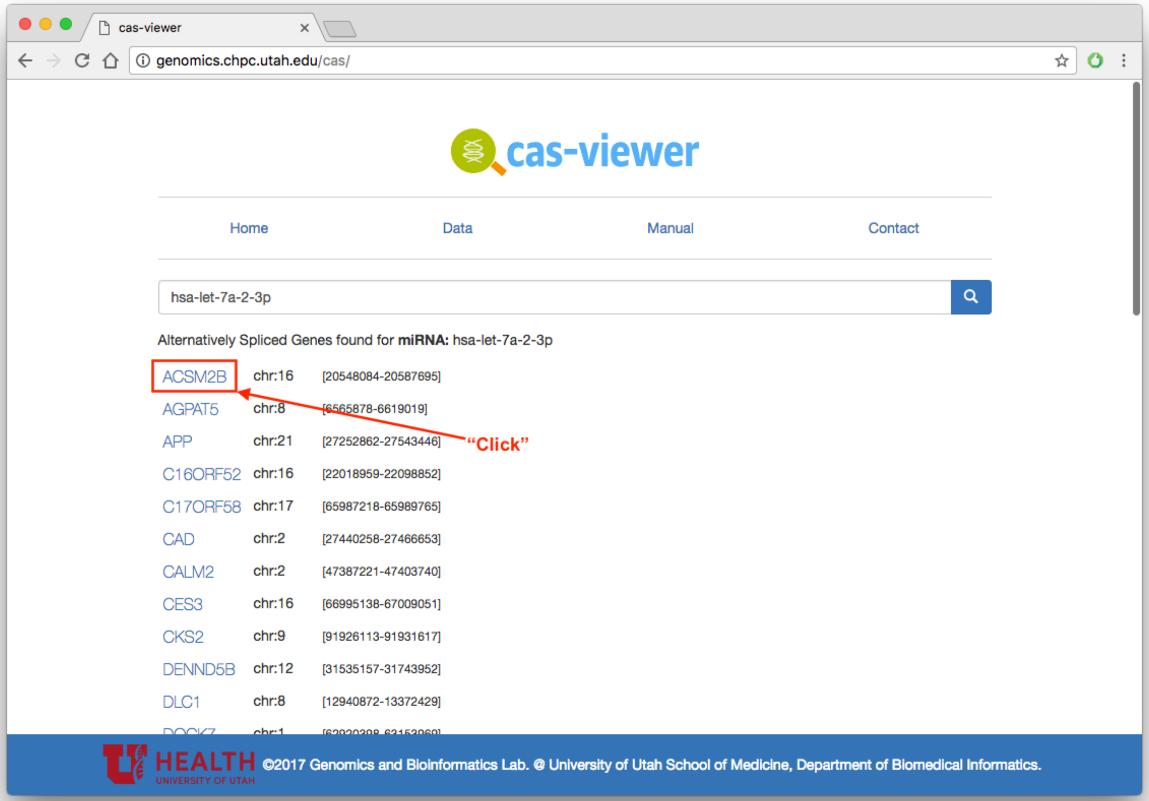

Figure 2. Search for AS genes of an miRNA (hsa-let-7a-2-3p)

3. AS Gene Navigator

3.1 AS Gene Viewer

Figure 3 is a screenshot of AS Gene Viewer for the ACSM2B gene, which is seen after clicking "ACSM2B," as shown in Figure 2. This gene has three transcript isoforms, comprising 16 distinct exons in total. The block and line represent the exon and intron (**Figure 3a**). Among exon blocks, thicker blocks are the coding region (CDS), and thinner blocks are untranslated regions (UTRs) (**Figure 3a**).

3.2 Exon Usage Track

The transcript track named "Exon usage" on the left side is composed of the representative exons that are defined by clustering overlapping exons. Exons are clustered according to the genomic location to find overlapping exons. Then, we create "representative exons," which is essentially a concatenation of the longest exons in each exon cluster. Exons whose length differs from the representative exon can be recognized easily to be alternatively spliced (i.e., 5' and 3' splice sites and intron retention). The color on the exon represents the skipping frequency of each exon; the lighter the color, the more frequently it is skipped. The user can also see the same information through the mouse over pop-up for each exon that shows how many transcripts miss the given exon.

3.3 Methylation, miRNA, and SRE SNP Track

SRE SNPs, methylations, and miRNAs that exist within the defined transcribed region of a gene are visualized accordingly (**Figure 3a**).

3.4 Intron Scaling

An intron scale of 0% is a transcript viewer in the mRNA coordinate that indicates splicing features (**Figure 3a**). An intron scale of 100% makes the viewer e equivalent to a genome browser, which is convenient for specifying the genomic features in introns (**Figure 3b**).

3.5 Select an exon

When you click an exon of interest, the selected exon is highlighted in green (**Figure 4a**). The exon selection in AS Gene Navigator pre-divides the transcripts into two groups: transcript(s) with exon skipping and transcript(s) without exon skipping as a default. The two groups are by default in the 'Group a Transcript' Option, but the user can re-group the transcripts under Option (**Figure 4b**).

a.

b.

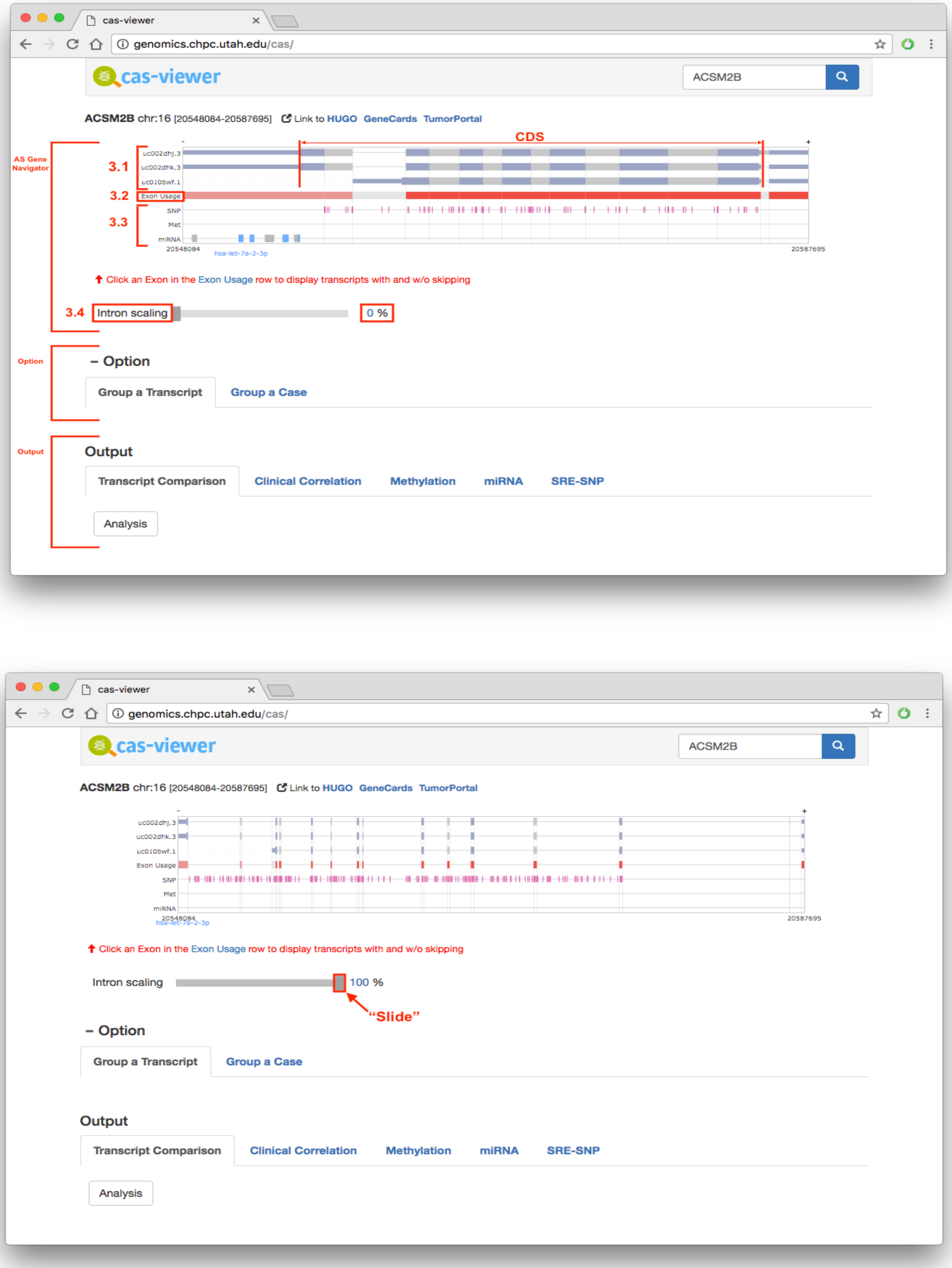

Figure 3. Screenshot of AS Gene Navigator for *ACSM2B* gene. (a) Transcript viewer with intron scaling of 0%. (**b**) Genome viewer with intron scaling of 100%.

a

b

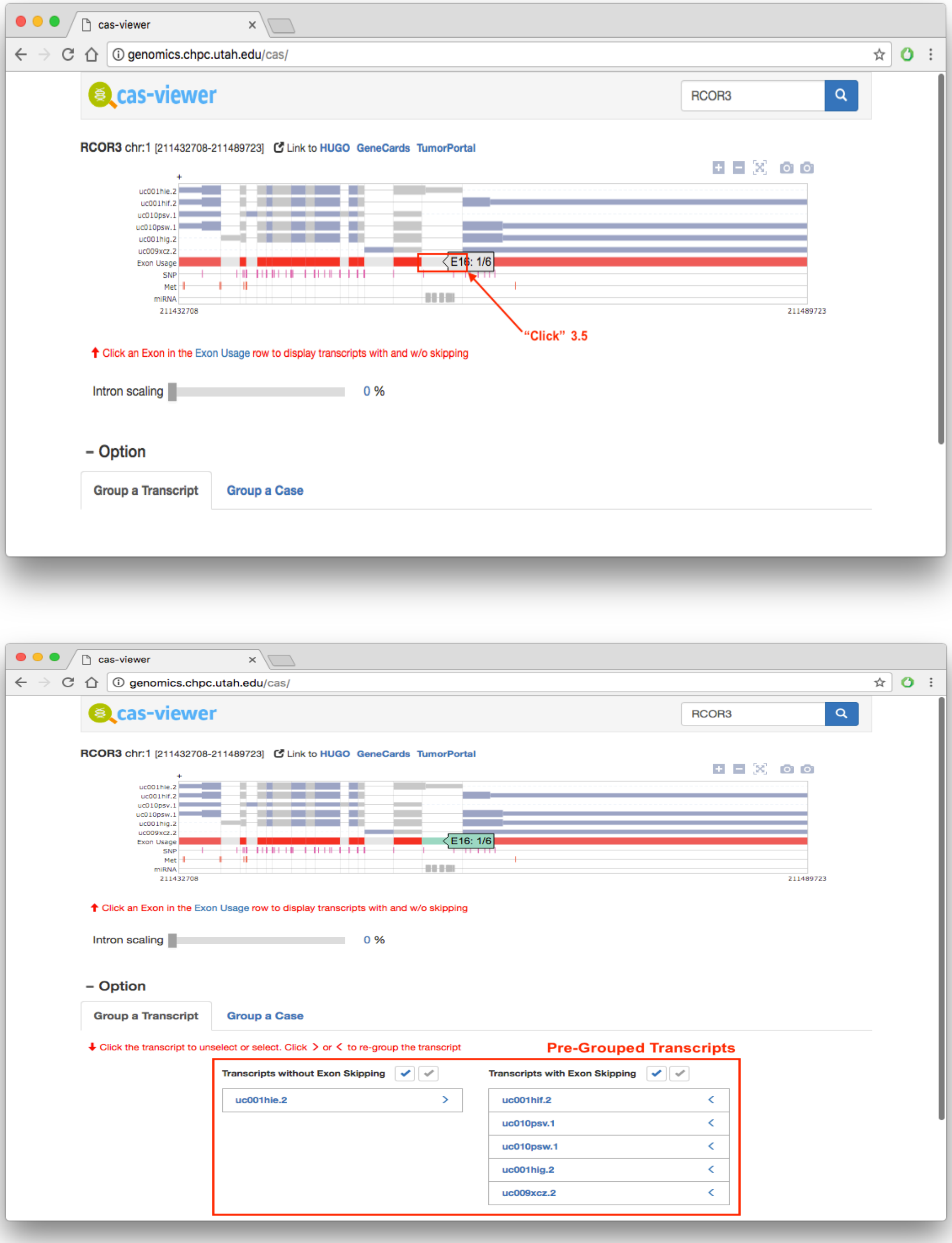

Figure 4. Select an exon. (**a**) One exon selection and (**b**) pre-grouped transcripts according to skipping of selected exons.

4. Option

4.1 Group a Transcript

Once the user selects an exon of interest in AS Gene Navigator, by default, the transcripts are automatically pre-divided into two groups: transcript groups with and without skipping of the selected exon (**Figure 4b**). Then, the user can further re-group the transcripts by 1) clicking the transcript id to unselect the pre-selected transcript and 2) clicking ">" or "<" to move the transcript into another group. **Figure 5a** and **Figure 5b** show the re-grouping options and regrouped transcripts, respectively. The ratio of differential expression (denoted "percent spliced in" (PSI)) between the two transcript groups will be calculated and plotted in "Transcript Comparison" of the output panel. The PSI value will be used for correlation test with clinical data, methylation data, and miRNA data.

a

b

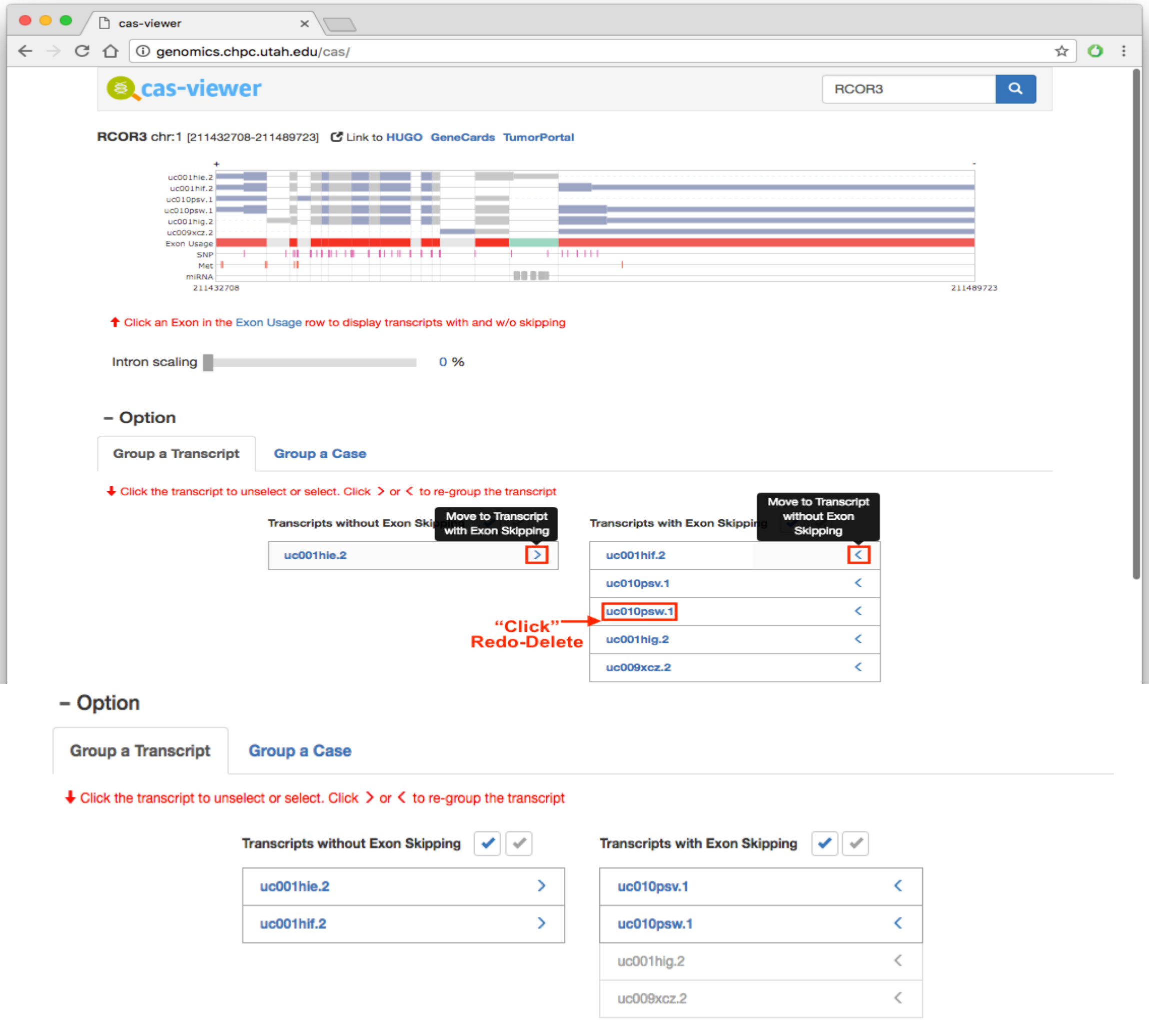

Figure 5. Option: Group a Transcript. (**a**) Using re-grouping options, (**b**) pre-divided transcript groups can be re-grouped with redo-selection within a group, and a transcript can be moved between groups.

4.2 Group a Case

The user can select a case according to its clinical information for a cancer type (**Figure 6**). Thirty-three cancer types are available in the option (**Supplementary Table 1**). The user defines two groups of cases within a cancer type to compare the clinical features to test whether the PSI correlates with expected survival times (**Figure 7**). This option helps users obtain evidence on which transcript isoforms (or exons) are important to the clinical outcome.

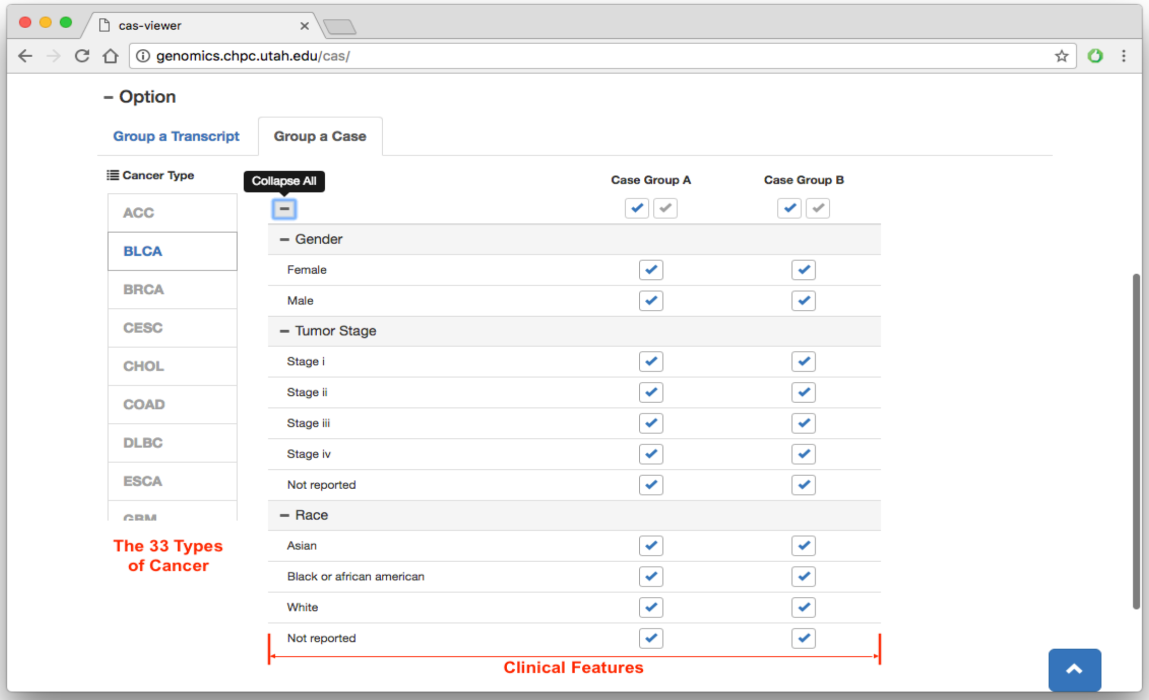

Figure 6. Option: Group a Case. The left panel lists 33 cancer types. Once a user selects a cancer type, the right panel shows the clinical features of the selected cancer, allow the user to select two cases to be tested in the Output.

a

b

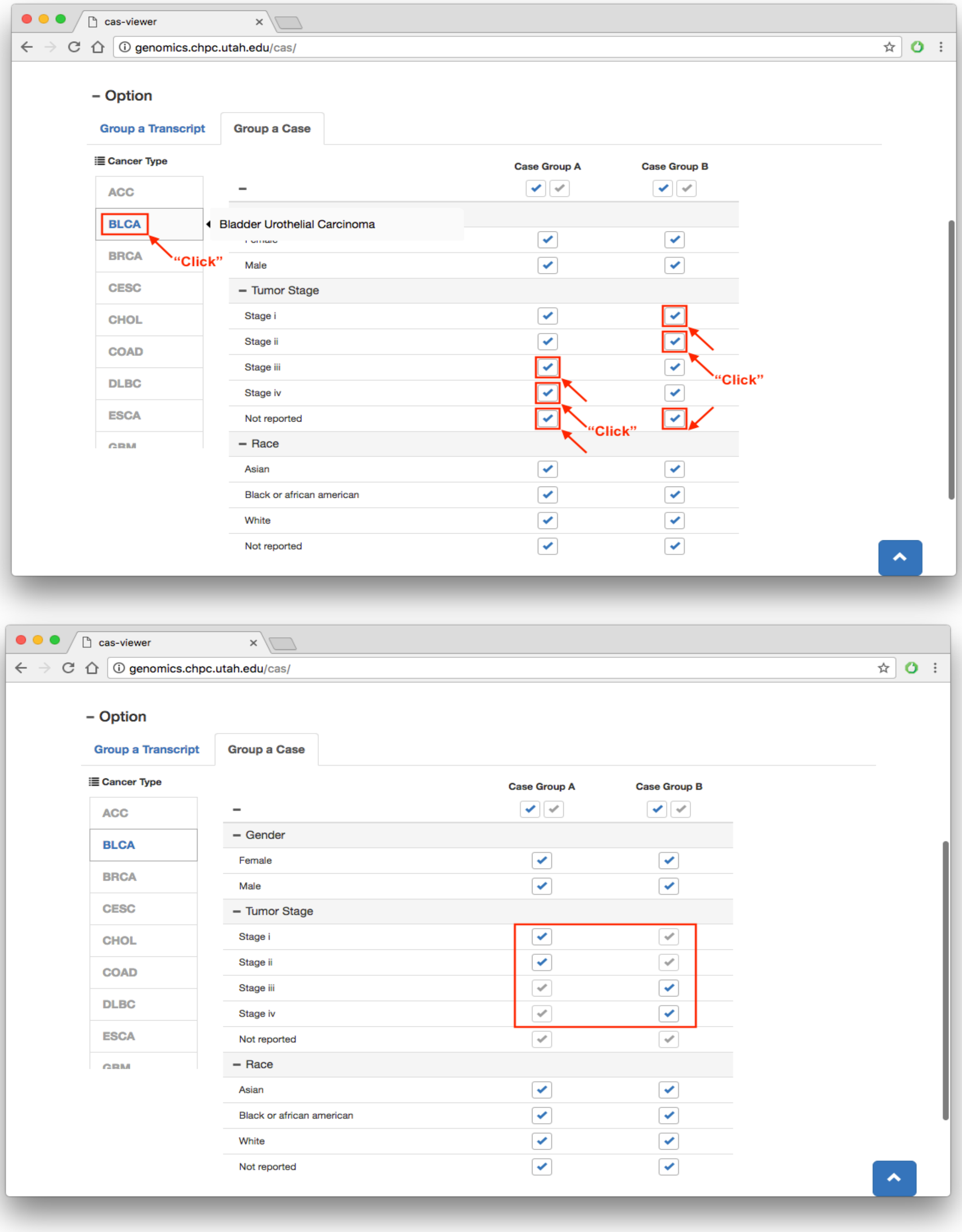

Figure 7. Example of two groups of cases selected in a demonstration of Output analysis. (**a**) In the left panel, the use clicks one cancer type and then clicks clinical features in 'case inclusion' option. (**b**) Within each clinical category, the "OR" option is selected, and between clinical categories, the "AND" option is selected to group a case.

5. Output

Output comprises five components for plotting the results: Transcript Comparison, Clinical Correlation, Methylation, miRNA, and SRE SNPs.

5.1 Transcript Comparison

The results are illustrated in the boxplot, showing the distribution of PSI values between two groups of cases, which is user-defined in the "Group a Case" Option. The PSI value refers to the differential expression between two groups of transcripts. In **Figure 8**, the X-axis is PSI value, ranging from 0 to 1, and the Y-axis is the two groups of cases. The plot shows the mouse over pop-up for each dot, giving detailed clinical information on each case; the p-value between differential PSI values between two groups of cases is shown at the bottom right. Under the plot, the table summarizes the number of cases for each group and the common cases in both groups, followed by the selected clinical features for each group.

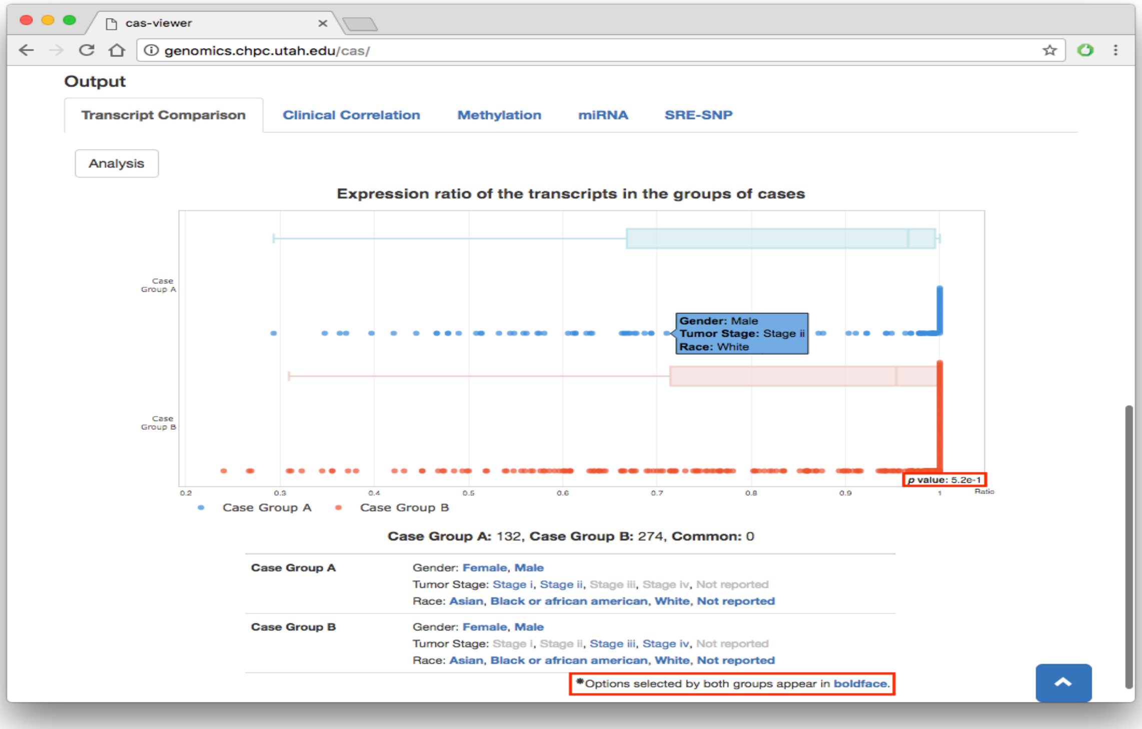

Figure 8. Transcript comparison

5.2 Clinical Correlation

For two groups of cases, this section summarizes the Kaplan-Meier plots, showing the correlation of PSI values with survival outcomes for three subgroups of cases: Case Group A, Case Group B, and Case Group B with high and low PSI. Then, the pair of high- and low-PSI subgroups for each Case Group is tested for significance of the differential survival outcome (**upper panel of Figure 9a**). The X-axis is expected survival time in days, and the y-axis is the survival ratio. In addition, the survival differences between Case Groups A and B are shown (**bottom panel of Figure 9a**). In the upper top of the plot, each legend is clickable, with the corresponding plot appearing and disappearing (**Figure 9b**). The dot for each survival time point displays further information on the mouse over pop-up.

a

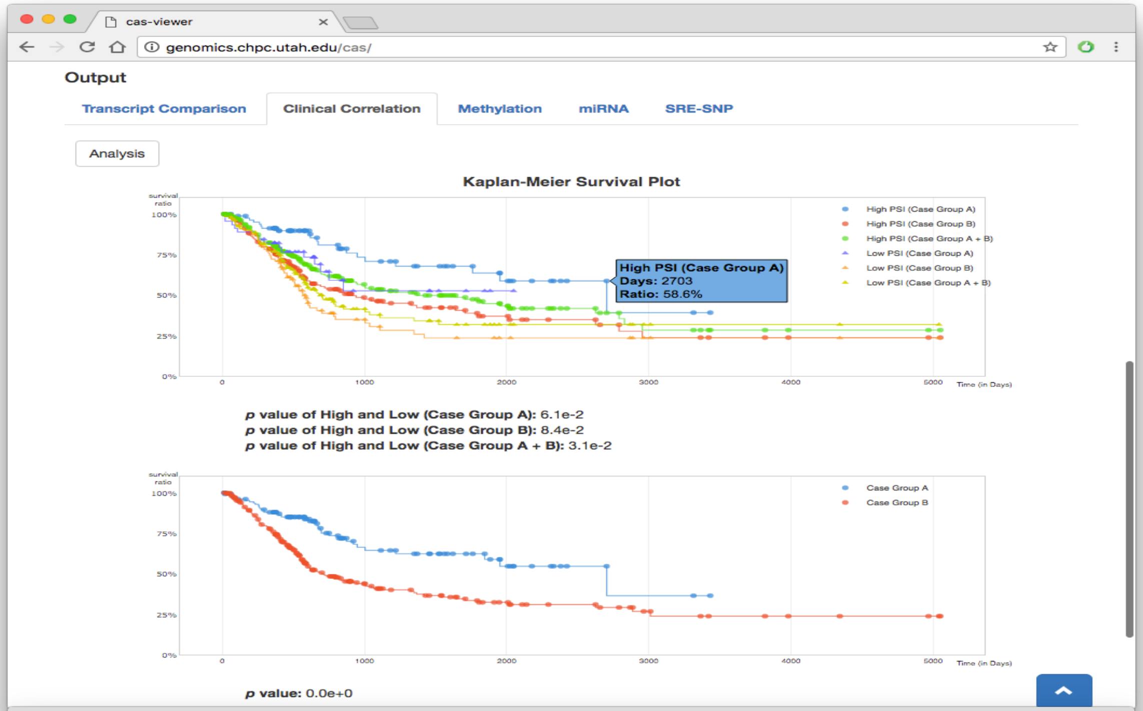

b

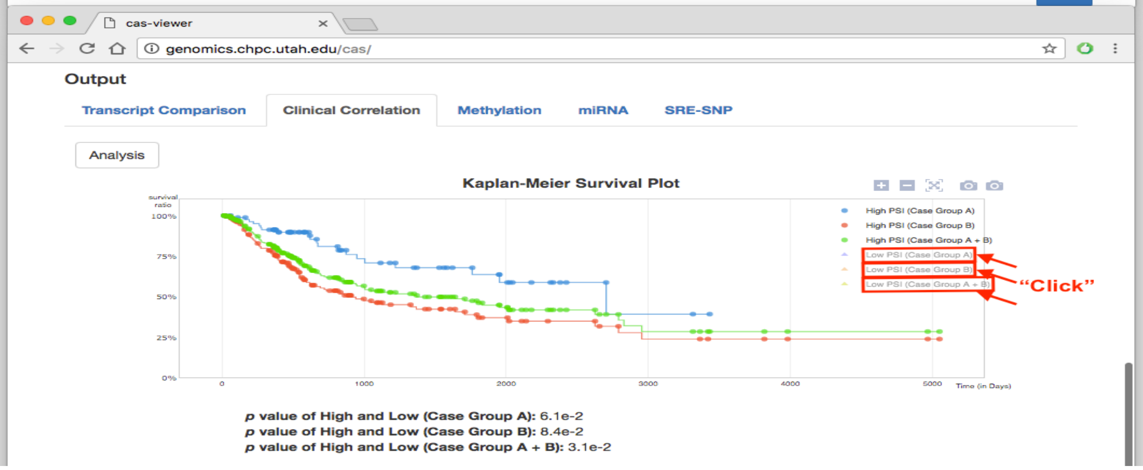

Figure 9. Clinical correlation of exon 16 in the RCOR3 gene

5.3 Methylation

Once a user clicks a certain exon in AS Gene Navigator, this panel automatically displays the zoomed-in view of the selected exon, including the right (downstream) and left (upstream) adjacent introns and all methylation that exists within the displayed genomic region. When a user clicks a point of methylation and the "Analysis" button, the plot summarizes the regression analysis by showing the distribution of cases according to PSI values on the y-axis and methylation status on the x-axis (**Figure 10a**). The mouse over pop-up on the dot displays the selected clinical features for a case. If a case is common in two groups of cases, the dot is green in the plot. At the bottom of the plot, the names of groups (i.e., Case Group A and Case Group B) are clickable, appearing and disappearing with the corresponding distribution of cases in the plot area (**Figure 10b**).

a

b

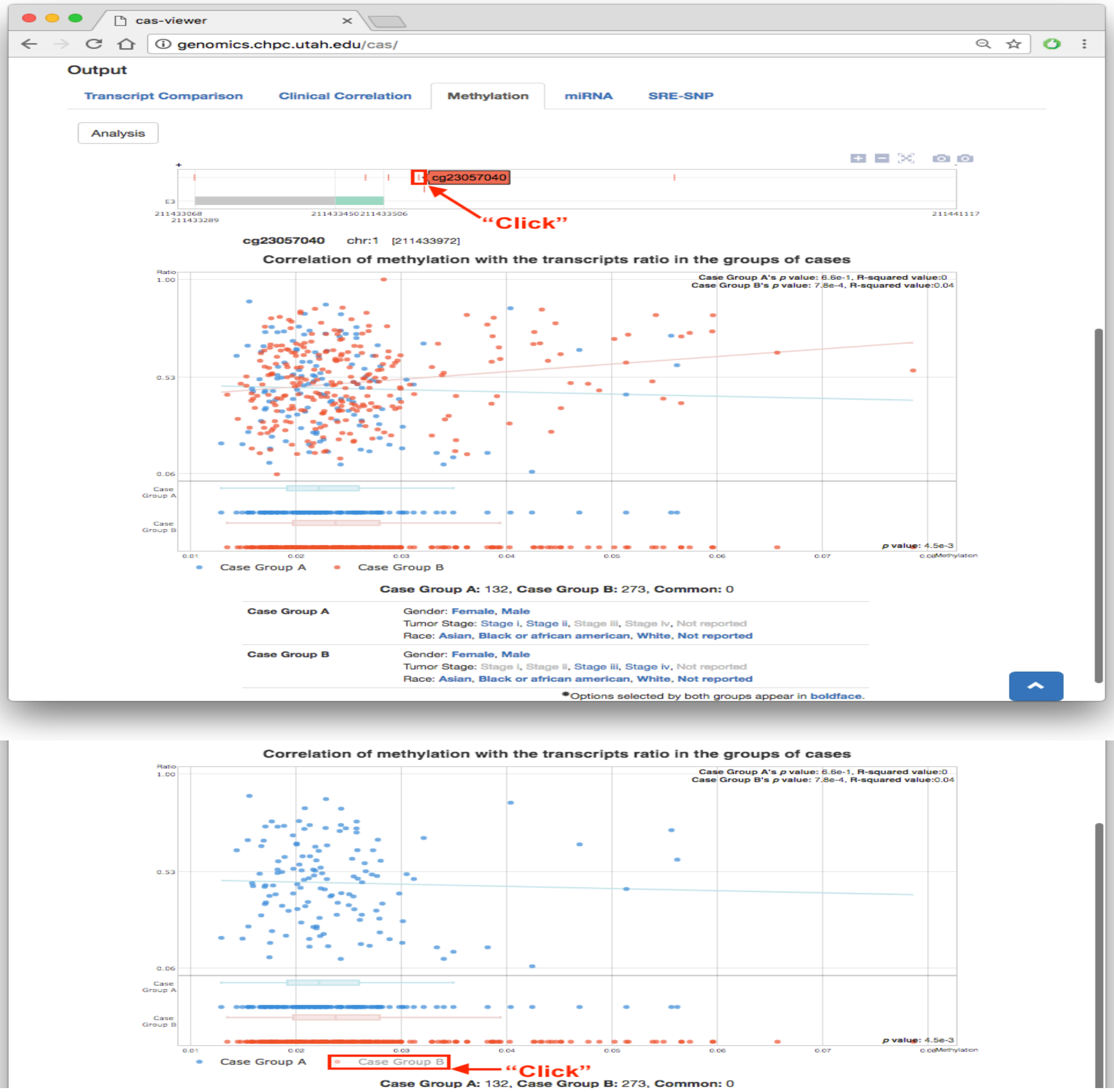

Figure 10. Methylation results for cg23057040 of exon 3 of the RCOR3 gene.

5.4 miRNA

Once a user clicks a certain exon in AS Gene Navigator, this panel automatically displays the zoomed-in view of the selected exon and all miRNA binding sites that exist within the displayed genomic region. When a user clicks a miRNA and the "Analysis" button, the plot summarizes the regression analysis by showing the distribution of cases according to PSI values on the y-axis and miRNA expression on the x-axis (**Figure 11a**). The mouse over pop-up on the dot displays the selected clinical features for a case. If a case is common in two groups of cases, the dot is green in the plot. At the bottom of the plot, the names of groups (i.e., Case Group A and Case Group B) are clickable, appearing and disappearing with the corresponding distribution of cases in the plot area (**Figure 11b**).

a

b

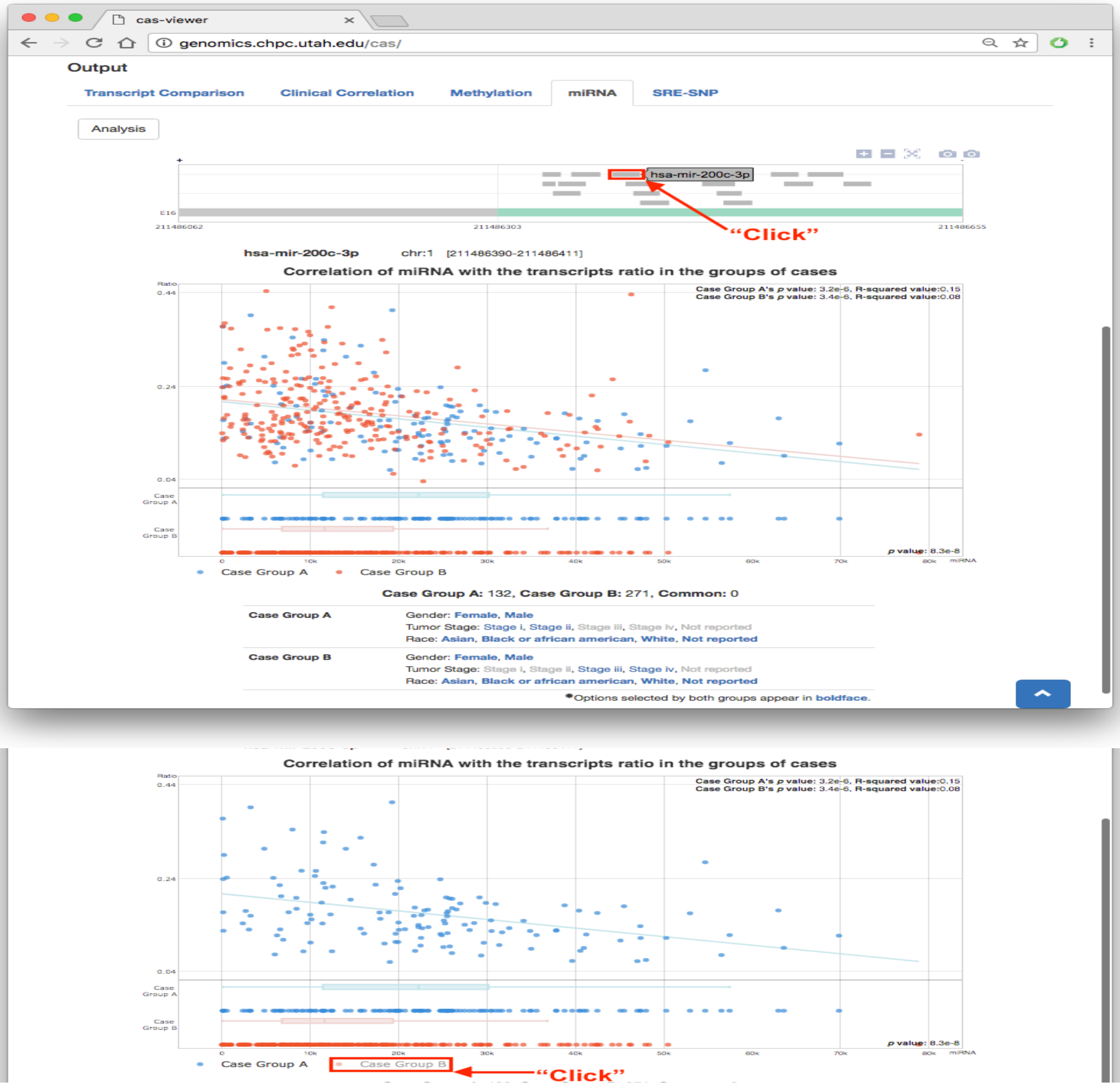

Figure 11. miRNA results for hsa-mir-200c-3p in exon 16 of the RCOR3 gene

5.5 SRE SNP

The genomic regions showing SRE SNPs for a selected exon comprises the right and left introns adjacent to the exon and selected exon. When one selects an SNP, this component summarizes the hexameric SRE motifs (i.e., ESE, ESS, and ISE) in the SNPs.

As an example, for rs184034715, the 'SRE-SNP' tab shows the zoomed-in view of the selected exons and introns as defined above with SRE SNPs. Clicking the SNP displays all predicted SRE information, including genomic location and hexameric sequences (**Figure 12**).

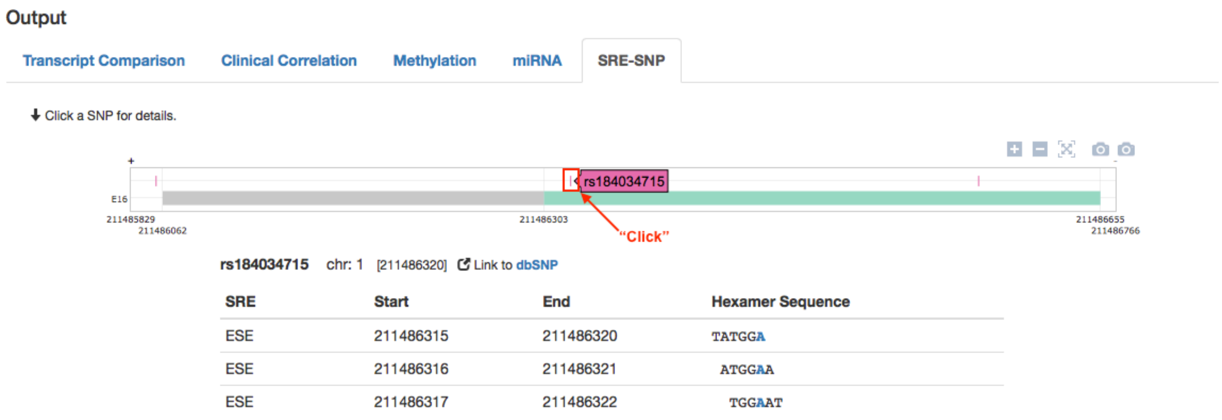

Figure 12. SRE SNP results for rs184034715 in exon 16 of the RCOR3 gene.

References

Genomes Project, C., *et al.* A global reference for human genetic variation. *Nature* 2015;526(7571):68-74.

Han, S., Jung, H., Lee, K., Kim, H., Kim S. Genome wide discovery of genetic variants affecting alternative splicing patterns in human using bioinformatics method. *Genes Genom* 2017;39(4):453–459.

Kent, W.J. BLAT - The BLAST-like alignment tool. *Genome Research* 2002;12(4):656-664.

Lee, Y., *et al.* Variants affecting exon skipping contribute to complex traits. *PLoS Genet* 2012;8(10):e1002998.

Therneau, T. A package for survival analysis in S. (R package version 2.36–12, 2012).

Yeo, G., *et al.* Variation in sequence and organization of splicing regulatory elements in vertebrate genes. *Proc Natl Acad Sci U S A* 2004;101(44):15700-15705.
